# Supplementary figures and images for: Genetic architecture of transmission stage production and virulence in schistosome parasites
Source: Virulence. 2021 Jun 24;12(1):1508–26. doi: 10.1080/21505594.2021.1932183 (PMC8237990; doi:10.1080/21505594.2021.1932183)

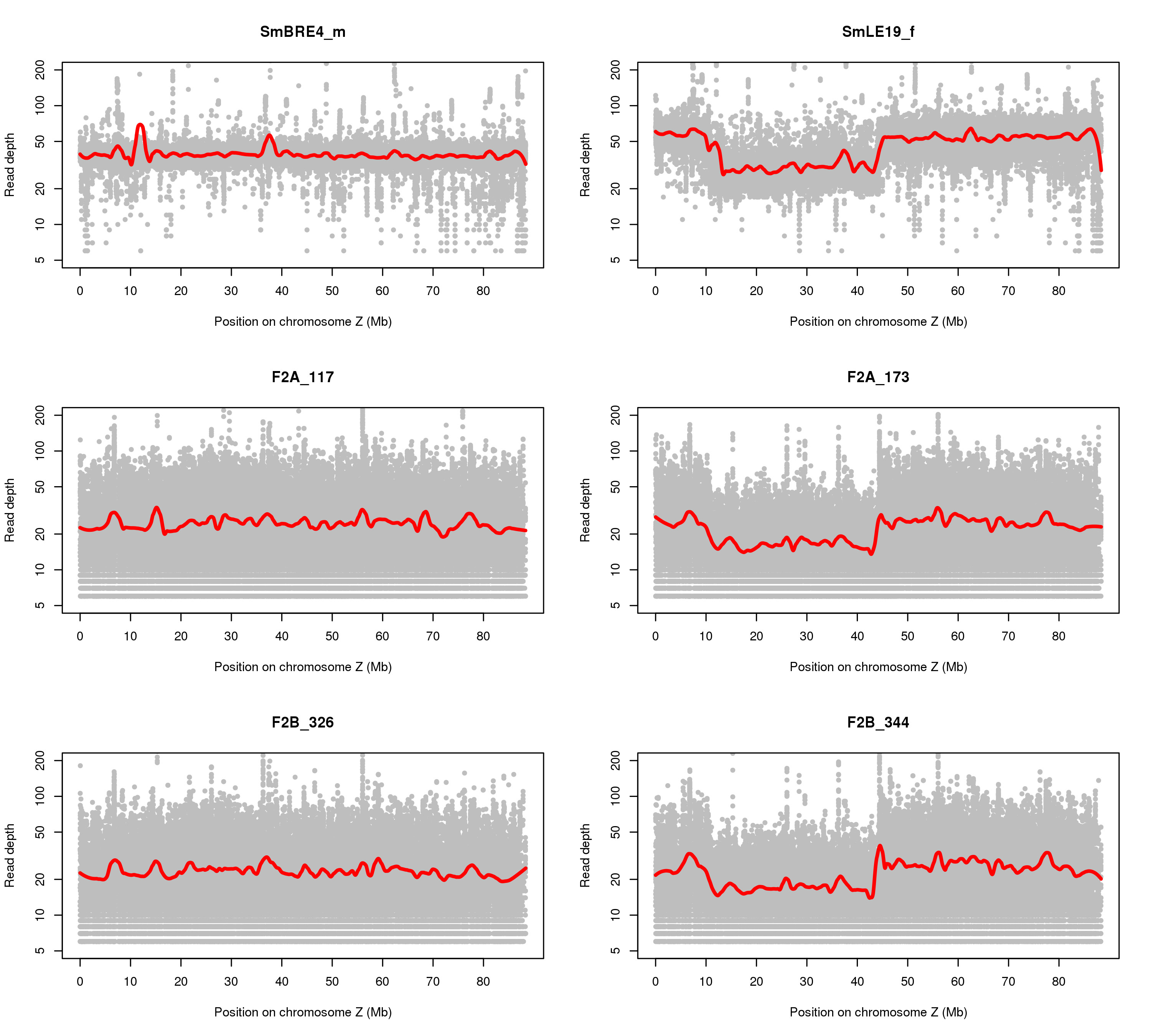

Supplement: Supplemental Material [file KVIR_A_1932183_SM5056.zip › supplementary/Supplementary_figure1.jpeg]

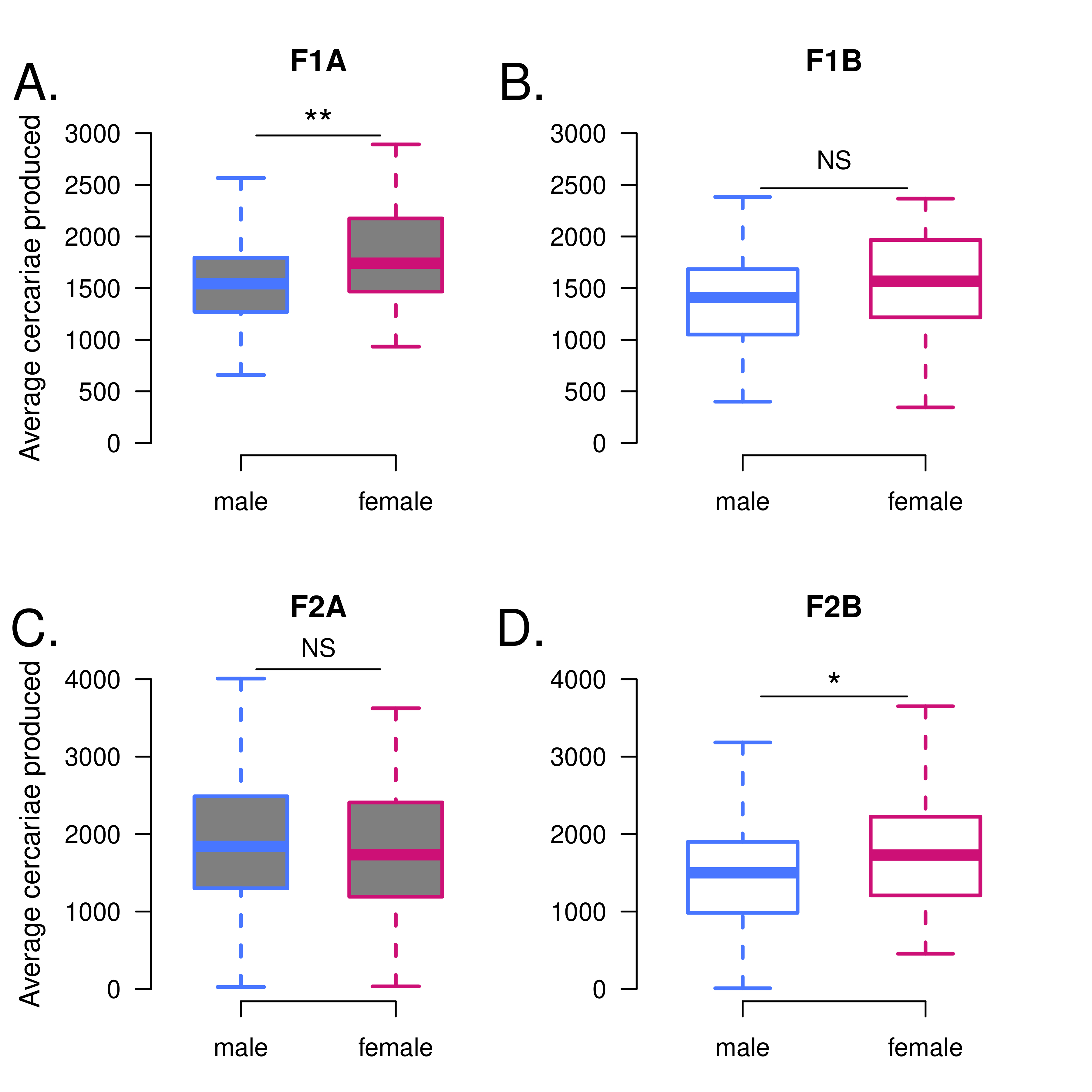

Supplement: Supplemental Material [file KVIR_A_1932183_SM5056.zip › supplementary/Supplementary_figure2.jpeg]

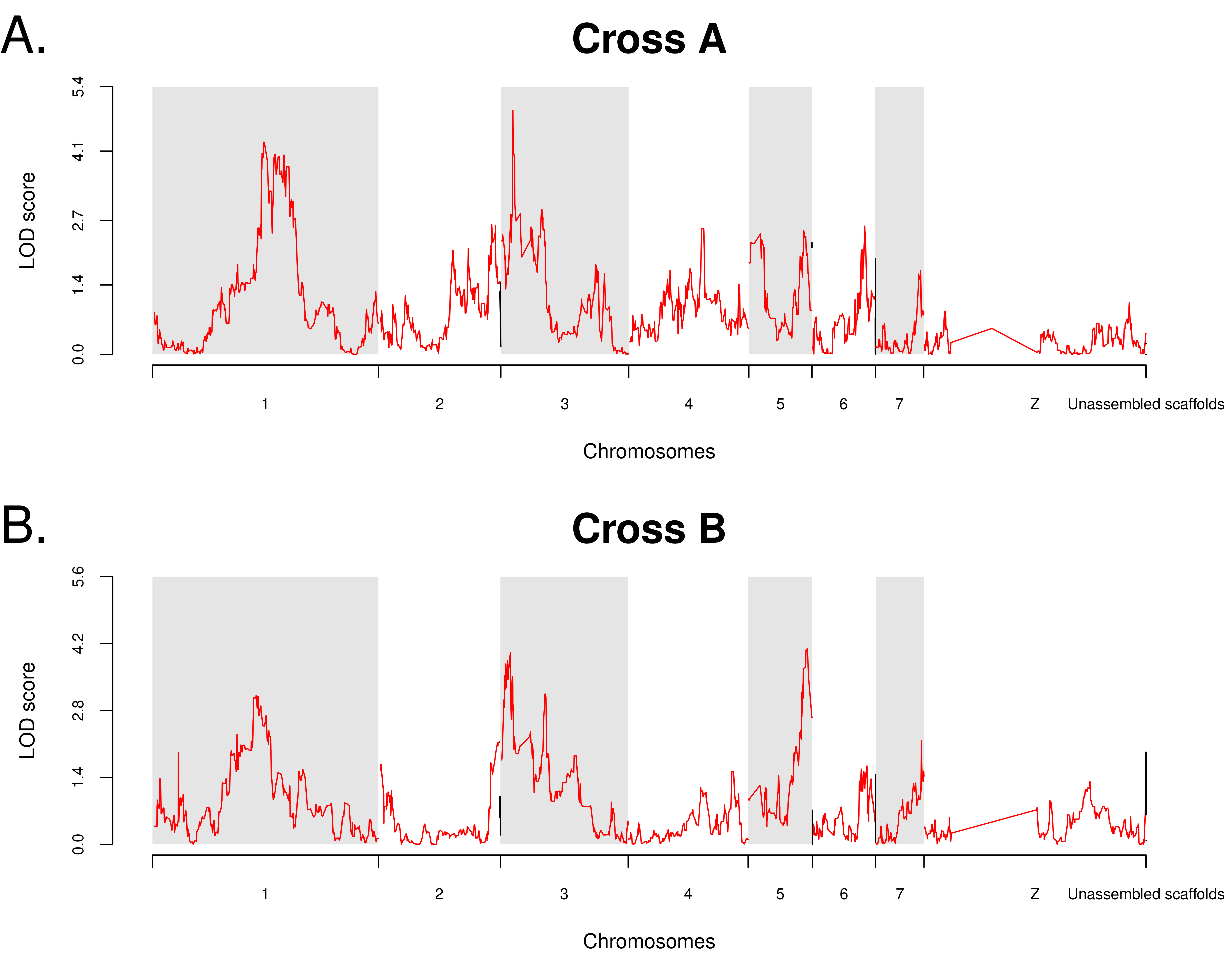

Supplement: Supplemental Material [file KVIR_A_1932183_SM5056.zip › supplementary/Supplementary_figure3.jpeg]

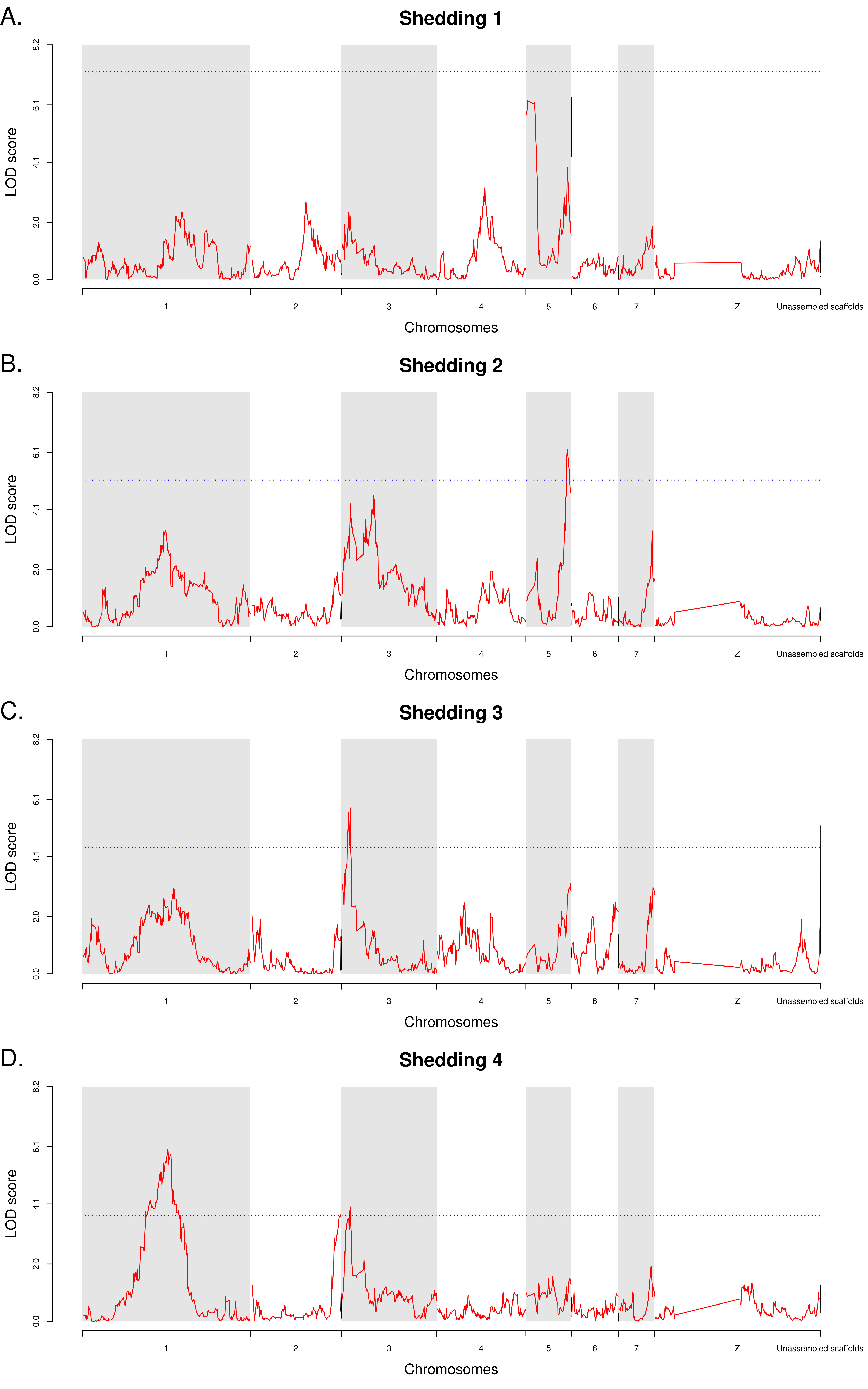

Supplement: Supplemental Material [file KVIR_A_1932183_SM5056.zip › supplementary/Supplementary_figure4.jpeg]

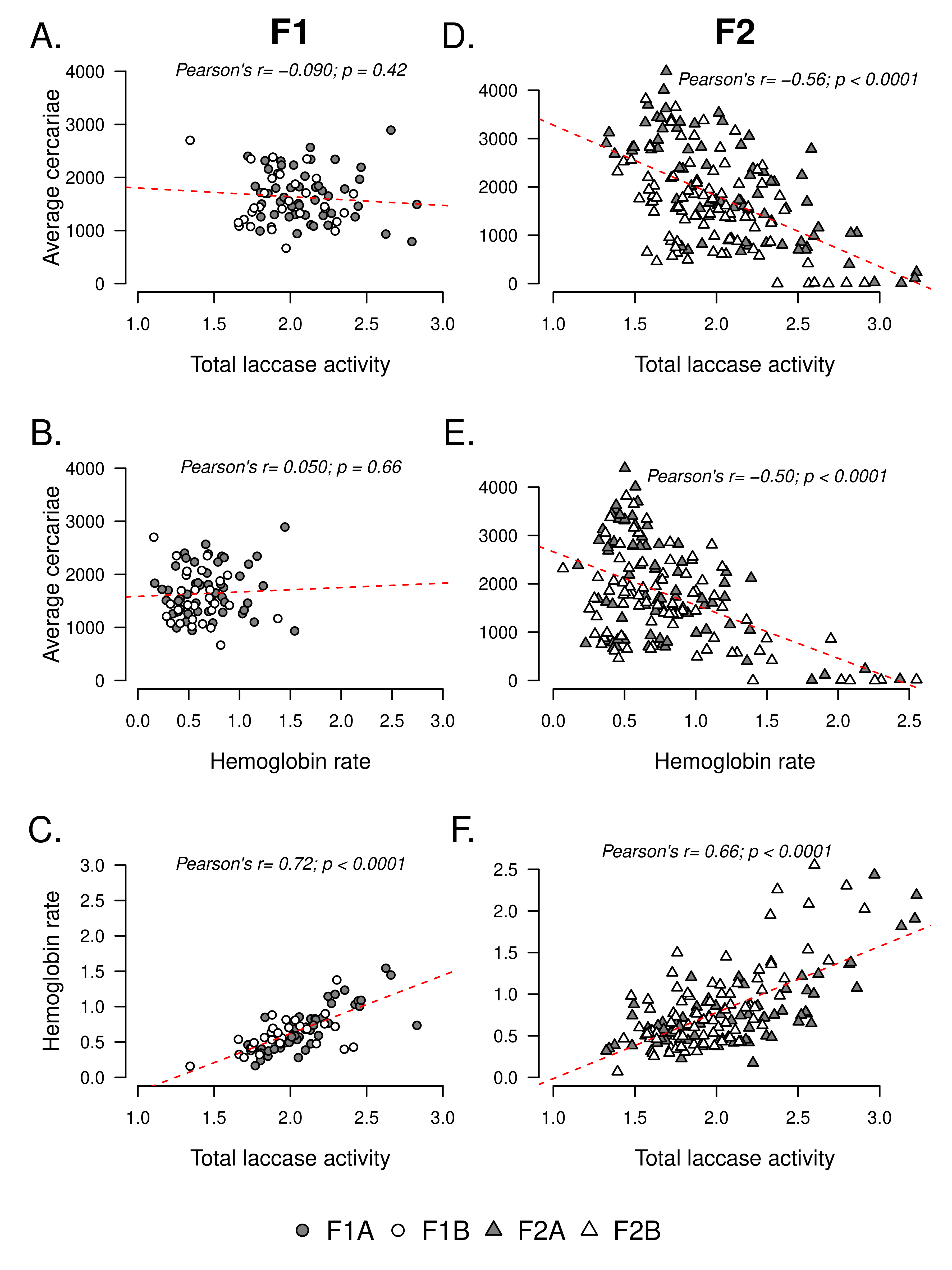

Supplement: Supplemental Material [file KVIR_A_1932183_SM5056.zip › supplementary/Supplementary_figure5.jpeg]
